# Novel Dutch Self-Assessment Biosecurity Toolkit to Identify Biorisk Gaps and to Enhance Biorisk Awareness

**DOI:** 10.3389/fpubh.2014.00197

**Published:** 2014-10-20

**Authors:** Petra C. C. Sijnesael, Linda M. van den Berg, Diederik A. Bleijs, Paul Odinot, Carin de Hoog, Mieke W. J. C. Jansen, Evelien Kampert, Saskia A. Rutjes, Martien Broekhuijsen, Sander Banus

**Affiliations:** ^1^Biosecurity Office, Centre for Environmental Safety and Security, National Institute for Public Health and the Environment, Bilthoven, Netherlands; ^2^Stout Groep BV, Echteld, Netherlands; ^3^Genetically Modified Organisms Office, Centre for Safety of Substances and Products, National Institute for Public Health and the Environment, Bilthoven, Netherlands; ^4^Corporate Real Estate and Campus, Taskforce Safety and Environment, Utrecht University, Utrecht, Netherlands; ^5^Service Organisation, Human Resources, Erasmus MC, University Medical Centre Rotterdam, Rotterdam, Netherlands; ^6^Staff Unit Compliance, National Institute for Public Health and the Environment (RIVM), Bilthoven, Netherlands; ^7^Centre for Infectious Disease Control, National Institute for Public Health and the Environment, Bilthoven, Netherlands; ^8^Marble ChemBio Consulting BV, Schipluiden, Netherlands

**Keywords:** biosafety, biosecurity, biorisk, CWA15793, questionnaire, toolkit, awareness, self-assessment

## Introduction

Life sciences, biotechnology, and medical biology are indispensable research fields for public health and the development of therapeutics and vaccines. However, biological agents and information developed to better health, welfare, and safety, could be misused for harmful purposes to cause damage to public health, safety, and the environment (1–3), which is termed the “dual-use” aspect of research in the life sciences. Laboratory biosafety describes containment principles, technologies, and practices to protect people from biological agents, and prevent accidental release of biological agents (4). In addition to biosafety, laboratory biosecurity measures aim to prevent theft and intentional or malicious use of biological agents (4). Thus, both biosafety and biosecurity should be an integral part of program management of organizations handling dangerous pathogens, in order to prevent potential dual-use research, undesired spread, theft, malicious use, and bioterrorism.

### From bioterrorism to biosecurity

The biosecurity program of organizations should contain physical, personnel, transport, technology, and material security (5). In addition, personnel should be well educated and aware of the biorisks of handling dangerous pathogens (1, 4–6). Theft and malicious or terrorist use of biological agents could possibly be traced back to breaches or lacunas in the biosecurity program of an organization. The next three historical examples of malicious use of biological agents illustrate the importance of biosecurity measures within organizations. The first example is the intentional spread of *Salmonella typhimurium*, which led to more than 750 cases of gastroenteritis in Oregon, USA, 1984. Members of the Bhagwan Shree Rajneesh commune ordered *Salmonella* bacteria from a commercial supplier, cultured the bacteria in their laboratory, and contaminated 10 salad bars (7). Criminal investigation revealed that the *Salmonella* outbreak strain was indistinguishable from the strain that had been cultured in the laboratory at the commune. The source of the biological material was a legitimate, easily accessible source, which underlines the importance of biosecurity awareness and the proper functioning of the biosecurity program of organizations. The second example of malicious use of biological agents is a biological attack in Japan. The Japanese religious cult Aum Shinrikyo tried to produce large-scale botulinum toxin and spores of *Bacillus anthracis*. The members isolated harmless strains of *Clostridium botulinum* from soil and thereby failed to produce active botulinum toxin in 1990 (8). For the production of anthrax, the members unsuccessfully attempted to steal *B. anthracis* from a laboratory. Later, the cult received anthrax from an Aum Shinrikyo sympathizer that had access to the biological agent within a university (8). However, this was an animal vaccine strain of anthrax, and not causing disease during dissemination, in 1993 (8). Thus, biosecurity pillars such as physical security, personnel screening, and personnel reliability are important in preventing theft of biological agents and bioterrorism. The last example describes the anthrax letters containing spores of *Bacillus anthracis* in 2001 in the USA. In total, 22 people were infected of which 5 people died. The source of the biological agent was a state laboratory involved in the national biodefense program of the USA (9). In addition to personnel screening and personnel reliability, material control and accountability might play an important role in preventing future malicious use of biological agents.

### Dutch biosecurity initiatives

The Dutch government recognizes the need to reduce biological threats and to prevent malicious use of biological agents. Therefore, the Dutch government and the Royal Dutch Academy of Arts and Sciences (KNAW) published the “Code of Conduct for Biosecurity” in 2007 (10). The code is intended to guide organizations and professionals that are, directly or indirectly, engaged in research or education in the life sciences, such as biology, medical biology, or biotechnology. Life sciences quickly evolve and new biosecurity and dual-use questions rise, such as the worldwide H5N1 biosecurity debate in 2011, 2012 (3). Therefore, the KNAW published the advisory report “Improving biosecurity, assessment of dual-use research” in 2013 (3). This report recommends biological threat analyses and an advisory board for research in the life sciences. Furthermore, the report emphasizes the importance of raising early awareness for the risks and potential misuse of research and knowledge in the life sciences.

In response to international biosecurity initiatives (11–15) and the evolving life sciences, the Dutch government initiated a biosecurity project to establish a coordinated Biosecurity Program for organizations handling hazardous biological agents and associated technology, in 2009. The purpose of this Biosecurity Program is to prevent proliferation of biological materials and associated knowledge for illegitimate purposes. As part of the Biosecurity Program, the Dutch Biosecurity Office was founded in 2012. The Biosecurity Office is the national knowledge and information center for biosecurity, and offers awareness raising workshops. The Biosecurity Office utilizes previously adopted good practices from both national and international initiatives, such as the BTWC, the EU CBRN Action Plan, CWA 15793, and the Dutch Biosecurity Code of Conduct. The Biosecurity Office cooperates with existing relevant organizations, such as the Dutch Platform of Biosafety Professionals. The biosecurity policy in the Netherlands reflects the current worldwide trend to combine biosafety and biosecurity into biorisk program management (4, 15).

### Online “biosecurity toolkit”

In close collaboration with the Dutch Platform of Biosafety Professionals and other experts, the Biosecurity Office developed the online “Biosecurity Toolkit,” in 2012 and 2013. The Biosecurity Toolkit aims at enhancing biorisk management within organizations handling hazardous biological materials. The Toolkit is a self-assessment tool that is freely available via www.biosecuritytoolkit.com in Dutch and in English. The Toolkit is an easily accessible tool for professionals and organizations to analyze gaps in their institutional biosecurity management. The outcome of the Toolkit includes best practices per biosecurity pillar to improve the biosecurity level of the organization. The use of the Toolkit is anonymous and online results are not stored. The Toolkit helps organizations to assess their current level of biosecurity and combines biosafety and biosecurity into biorisk.

## Methods

The Biosecurity Toolkit has specifically been developed for organizations handling hazardous or dangerous biological agents. Representatives from those organizations, governmental representatives, and biosafety/biosecurity experts were invited to participate in the development process of the Biosecurity Toolkit. This group of stakeholders and experts convened in several meetings to compose the toolkit and ascertain applicability of the Toolkit for the intended users.

### Questionnaire

**Figure 1:**
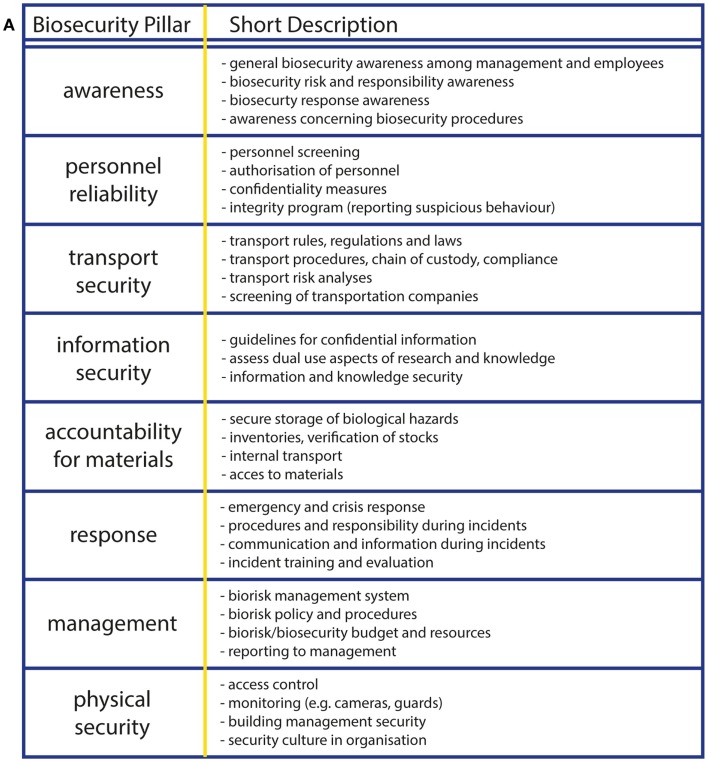

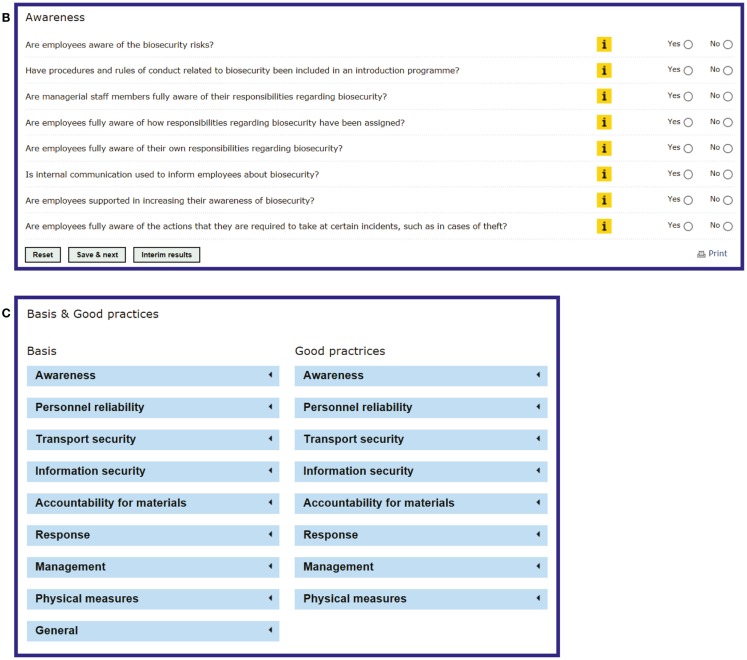
**The online Biosecurity Toolkit has eight biosecurity pillars**. **(A)** The eight biosecurity pillars were adapted from previous studies (5, 16) and were ascertained by the Dutch biosecurity expert group and biosecurity stakeholders. In the left column, the pillars are placed in the order of appearance in the online Toolkit. In the right column, a short description per biosecurity pillar is provided. **(B)** The pillars are placed in the tab pages on the top of the webpage where the questionnaire for “Awareness” is shown. By clicking on the subsequent pillar, the questions become visible and can be answered with “yes” or “no.” The yellow “i” information button provides information about the specific question. The online questionnaire can be saved between pillars, and interim results can be viewed at any convenient time. By clicking the “reset” button, the form will be cleared from previously entered answers. **(C)**. The tab page “Good Practices” contains legal bases and good practices for biosecurity program improvement. By clicking on specific biosecurity pillars, a list with links, best practices, and information is available with suggestions for improvement of the biosecurity program within organizations.

The experts defined eight pillars of biosecurity risk management, namely awareness, personnel reliability, transport security, information security, accountability for materials, response, management, and physical security (5, 6, 16). The biosecurity experts added the eighth pillar “management” to the Biosecurity Toolkit, since the management of an organization should also be aware of biological risks, and commitment of the higher management is a prerequisite for successful implementation of the biorisk management program. A short description per biosecurity pillar is provided in Figure 1A. Per biosecurity pillar, the user needs to answer up to 10 questions with “yes” or “no” in the questionnaire (Figure 1B), and the relative score for each category is normalized to 100%. In case of doubt or uncertainty, the user is advised to fill in “no,” so the associated suggestion for improvement will be addressed after fulfilling the Toolkit. Each question is accompanied with explanatory or background information, accessible via the information icon (Figure 1B). The questionnaire can be saved and interim results can be viewed between different pillars, at every convenient time for the user.

### Legal basis and good practices

Supplemental information about legal basis and good practices is provided under the tab page “Good practices” (Figure 1C). The information under “Basis” refers to national and international laws, guidelines, standards, and other relevant documents that are available in the Netherlands, such as the Biosecurity Code of Conduct (10) and CWA 15793 (15). The column “Good Practices” lists specific biosecurity measures that may increase the biosecurity level of that particular biosecurity pillar. The good practices have been formulated in collaboration with experts from the field.

### Results section of the toolkit

After completing the questionnaire, the user is directed to the results section of the Toolkit and the outcome of the survey is automatically presented to the user. Relative scores for each category are calculated as a percentage (actual score as percentage of the maximum achievable score). Importantly, the overall score is not calculated as an average of the individual scores, but is equal to the *lowest* score obtained in the separate elements. The overall score is presented as lowest score since the aim of the survey is to identify gaps and strengthen the biosecurity program, which is most effectively obtained by improving the weakest element in an organization.

## Examples and Conclusion

The type of organization, the biological agents handled by the organization, the risks associated with executing proceedings, the dual-use potential or likelihood that an agent can be misused, and many more variables are important for designing and implementing a biosecurity program within the organization (6, 16). To illustrate the use and possible gap analysis of the Biosecurity Toolkit, we hypothetically describe two types of organizations handling dangerous pathogens: a high-containment diagnostic laboratory from a university medical center, and a high-containment laboratory from a pharmaceutical company.

### High-containment diagnostic laboratory in a university medical center

For diagnosed or suspicious hazardous material, the university medical center has a BSL3 facility. Only authorized personnel are allowed to enter and conduct laboratory work in the BSL3 facility. Reference material and patient samples are stored within the containment of the BSL3 facility. Since the laboratory is part of a university medical center, knowledge is shared among different departments and potential hazardous samples may be used for research or scientific purposes. The employees are fully aware of biosafety risks; however, there is less awareness for biosecurity and dual-use risks of the samples. Entrance to the BSL3 facility has been restricted to authorized employees only, however, the medical center is a public, open organization and outsiders can easily enter the hospital. There is no security culture within the center. The medical center scores well on external transport security; however, internal transport of diagnostic samples that are used for scientific purposes is pore documented in procedures. The same applies for information security: the university medical center has guidelines for confidentiality of patient samples, but no guidelines for securing and following research samples. The hospital has procedures for emergency and crisis response, and has a clear policy of communication in case of emergencies. Thus, the fictive gap analyses for the medical center identified gaps for biorisk management system, physical measures, biosecurity awareness, and personnel reliability. The center scores well on material accountability and response, and scores average on information security and transport security.

### Laboratory in a pharmaceutical company

This pharmaceutical company develops vaccines against airborne influenza viruses. The company has BSL3 animal facilities and laboratories for research purposes. The company is located in a rural area and has strict entrance security. Employees are background checked and research is well documented, since patents and intellectual property are important for the development of vaccines. The organization has high standards regarding general security, biosafety regulations and well-documented research, recorded in standard operating procedures, and procedures describing coding of materials. The fictive gap analyses for the pharmaceutical company identified gaps for biosecurity awareness and response, specifically in case of dual-use research awareness. Although personnel are well educated and trained for handling dangerous airborne pathogens, this training has been focused on biosafety and not on biosecurity awareness. The same applies to response and incidents: response in case of biosafety incidents and theft have been documented in procedures and covered in the employee training; however, no procedures are present regarding emerging dual-use research. Thus, the company scores well on personnel reliability, physical measures, material accountability, and information security. The company scores less on response and biorisk management, since biosafety and biosecurity are not integrated in the company.

## Conclusion

Here, we describe an online self-assessment “Biosecurity Toolkit,” which was developed to strengthen awareness among laboratory employees, biosafety or biosecurity officers, the management team, or security managers of organizations handling dangerous biological agents. The web-based Biosecurity Toolkit offers a free and easily accessible tool and the resulting gap analysis of the questionnaire is for internal use only. The results are anonymous and not automatically uploaded or stored. The main purpose of the toolkit is to provide the user insight in the level of biosecurity within the organization, to create awareness and above all, to provide suggestions for improvement of the biosecurity level by focusing on the weakest elements.

## Author Contributions

Petra C. C. Sijnesael, Linda M. van den Berg, Diederik A. Bleijs, and Martien Broekhuijsen wrote the manuscript. Petra C. C. Sijnesael, Linda M. van den Berg, Diederik A. Bleijs, Paul Odinot, Carin de Hoog, Mieke W. J. C. Jansen, Evelien Kampert, Saskia A. Rutjes, and Martien Broekhuijsen investigated, designed, and tested the Biosecurity Toolkit. Sander Banus supervised all aspects and execution of the project. All authors critically reviewed the manuscript for correct content.

## Conflict of Interest Statement

The authors declare that the research was conducted in the absence of any commercial or financial relationships that could be construed as a potential conflict of interest.
